# Splash!: a prospective birth cohort study of the impact of environmental, social and family-level influences on child oral health and obesity related risk factors and outcomes

**DOI:** 10.1186/1471-2458-11-505

**Published:** 2011-06-27

**Authors:** Andrea M de Silva-Sanigorski, Elizabeth Waters, Hanny Calache, Michael Smith, Lisa Gold, Mark Gussy, Anthony Scott, Kathleen Lacy, Monica Virgo-Milton

**Affiliations:** 1 McCaughey Centre, Melbourne School of Population Health, The University of Melbourne, Carlton Australia; 2 Dental Health Services Victoria, Carlton, Australia; 3 Oral Health Services, Barwon Health, Geelong Australia; 4 Health Economics, Deakin University, Burwood Australia; 5 School of Dental Science, LaTrobe University, Bendigo Australia; 6 Melbourne Institute of Applied Economic and Social Research, The University of Melbourne, Carlton Australia; 7 WHO Collaborating Centre for Obesity Prevention, Deakin University, Geelong, Australia

## Abstract

**Background:**

Dental caries (decay) is the most prevalent disease of childhood. It is often left untreated and can impact negatively on general health, and physical, developmental, social and learning outcomes. Similar to other health issues, the greatest burden of dental caries is seen in those of low socio-economic position. In addition, a number of diet-related risk factors for dental caries are shared risk factors for the development of childhood obesity. These include high and frequent consumption of refined carbohydrates (predominately sugars), and soft drinks and other sweetened beverages, and low intake of (fluoridated) water. The prevalence of childhood obesity is also at a concerning level in most countries and there is an opportunity to determine interventions for addressing both of these largely preventable conditions through sustainable and equitable solutions. This study aims to prospectively examine the impact of drink choices on child obesity risk and oral health status.

**Methods/Design:**

This is a two-stage study using a mixed methods research approach. The first stage involves qualitative interviews of a sub-sample of recruited parents to develop an understanding of the processes involved in drink choice, and inform the development of the Discrete Choice Experiment analysis and the measurement instruments to be used in the second stage. The second stage involves the establishment of a prospective birth cohort of 500 children from disadvantaged communities in rural and regional Victoria, Australia (with and without water fluoridation). This longitudinal design allows measurement of changes in the child's diet over time, exposure to fluoride sources including water, dental caries progression, and the risk of childhood obesity.

**Discussion:**

This research will provide a unique contribution to integrated health, education and social policy and program directions, by providing clearer policy relevant evidence on strategies to counter social and environmental factors which predispose infants and children to poor health, wellbeing and social outcomes; and evidence-based strategies to promote health and prevent disease through the adoption of healthier lifestyles and diet. Further, given the absence of evidence on the processes and effectiveness of contemporary policy implementation, such as community water fluoridation in rural and regional communities it's approach and findings will be extremely informative.

## Background

Dental caries (decay) is one of the most common diseases affecting children [1,2], in Australia and elsewhere [3], developing very early in life with detrimental impacts [4,5]. More than one third of four-year-old children experience caries in their deciduous (primary) teeth and, alarmingly, the majority of this caries experience is in the form of untreated decay [1,2]. Tooth decay can cause pain, discomfort and difficulty eating, and untreated tooth decay can become so severe that children must undergo tooth extraction, usually under general anaesthetic.

In Australia alone, in 2007-2008 close to 23,000 operations were performed in hospitals on the teeth, gums and tooth sockets of 1- to 14-year-old children, and for most of these operations, children required general anaesthetic for multiple tooth extraction [6]. Dental treatment was the 5th most common cause of hospitalisation for 1 to 4 year olds in 1999-2000 [7] and the rate is increasing. In the State of Victoria, admission rates for dental cases that are considered preventable (ACSCs) have increased over the last seven years in all age groups. There was a 62% increase in dental admissions between 1997-98 to 2004-05, with the rate per 1,000 population increasing by 24% [8]. Dental ACSC admissions moved from the fifth to the second ranked ACSC over this time [8]. In addition, the burden of dental disease is not evenly distributed, with the highest prevalence rates observed for children from disadvantaged communities, particularly children who are less exposed to population level strategies such as community water fluoridation, or potent prevention oriented health systems. In Australia, these patterns are observed for children in rural parts of the country [8-12].

**Figure 1:**
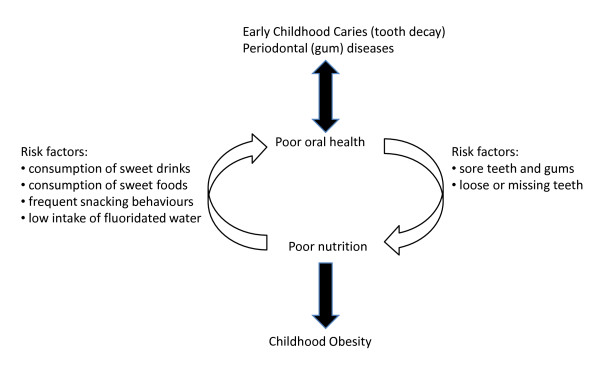
**Shared risk factors for poor oral health and childhood obesity**.

With population prevalence rates increasing by approximately 1% each year, overweight and obesity in childhood is also a public health concern. Depending on age, overweight and obesity affects between approximately one quarter and a third of Australian children, with poor health outcomes in childhood tracking through to adulthood, and the greatest burden experienced by those of lower socioeconomic background [13]. Common risk factors for both dental caries and childhood obesity are related to diet, as shown in figure 1, and the associations have been established between these diseases and a diet high in refined carbohydrates (predominately sugars), particularly the consumption of soft drinks and other sweetened beverages [14-18]. Conversely there is a demonstrable positive impact on healthy body weight and dental health if water is consumed in preference to other beverages [19,20]. However, comparatively little is known about the growing consumption of sweetened beverages and the health consequences of reductions in the consumption of alternative beverages (including water).

In 2005, the Victorian Child Health and Wellbeing Survey (VCHWS) included an item related to the consumption of tap water for the first time, with findings demonstrating that 50% of children residing in rural and regional Victoria were not drinking tap water[21]. In the Australian context, several key changes in water supplies have occurred over the past 10 years. Firstly, severe drought (and other environmental events) has led to changes in the source and/or taste of household water supplies. Secondly, fluoridation of household tap water supplies in rural and regional areas is being rolled out as a major dental public health initiative across Australia. Both these changes may lead to altered behaviours and patterns of consumption of tap water[22], compounded by population trends towards consumption of bottled waters (low or no fluoride and sometimes sweetened) or to a preference for fruit-based drinks and soft drinks (soda) that comprise high levels of sugars and acid [22].

Children from rural areas are three times more likely to be admitted to hospital for dental care compared to those from urban areas [23]. One factor that may make children living in rural areas particularly vulnerable to dental problems is the water environment to which they are exposed. This low intake of tap water may be related to the water environment, and drought and community water fluoridation may affect parents' perceptions of the safety and palatability of tap water [22]. This in turn may lead to changes in beverage consumption by the family, and particularly children. Parents may choose to provide their children with bottled water, sweetened flavoured water, fruit juice, fruit-based drinks or soft drinks in place of plain tap water.

Given that both dental caries and obesity are largely preventable and share common risk factors, the increasing incidence of both conditions demonstrates that sustainable and equitable solutions to address these significant geographical and socioeconomic health inequalities are essential. Although we have evidence that children from rural and regional areas are at greater risk of both dental decay and obesity, the actual pathways involved are not clearly articulated. There are a variety of influences on the behaviours that lead to the development of both disorders, however sugar intake and water consumption are common determinants. A significant evidence gap has been knowledge of the drivers of beverage consumption within the contemporary water and food policy context, particularly the role of socio-environmental, socio-cultural and socio-behavioural influences.

This study aims to:

1. Identify the determinants of parental choices concerning children's beverage consumption

2. Examine the importance of fluoridation and drought on perceived water quality and on beverage choice

3. Determine the impact of beverage choice on child obesity risk and oral health status

4. Compare these relationships for those living in rural and regional communities

Using the common risk factor [25,26] and life course frameworks, a mixed methods approach with qualitative research methods will be used to answer the first aim, and a prospective longitudinal cohort design to answer the second. The cohort will comprise infants (and their parents) from newly fluoridated and non-fluoridated areas of drought affected regional and rural Victoria.

The study objectives are:

1. To describe the attitudes and perceptions of parents towards water quality and beverage choice for their child(ren)

2. To determine the consumption pattern of beverages by young children and their parents/carers, the influence of parent drink choices on child beverage consumption and changes in consumption patterns during the first four years of the child's life

3. To measure the reach of fluoridation of public water supplies

4. To quantify the importance of perceived water quality, water fluoridation, water costs and other identified factors affecting drink choice

5. To determine the effects of water fluoridation and drought on drink choice and subsequent child health outcomes, particularly risk of poor oral health and obesity

6. To evaluate the impact of water fluoridation on inequalities of child oral health and obesity

## Methods/Design

This is a two-stage study using a mixed methods research approach to examine the effects of changes in the water environment, especially drought and community water fluoridation, on young children's oral and general health. The first stage involves qualitative interviews of a sub-sample of recruited parents to develop an understanding of the processes involved in drink choice and to develop measurement instruments to be used in the second stage. The second stage is a prospective cohort study where expectant mothers are recruited prior to the birth of their child, and children are followed prospectively over the next four years, with survey, biological, clinical and environmental data collected (Table 1). Baseline data will be collected from expectant mothers prior to the birth of the child, and data will be collected from mothers (primary carers) and children when the child is approximately six, 12, 24, 36 and 48 months of age.

**Table 1:** Study timeline, data collection and methods

	Child age (months)					
Measures	Pre-birth	6	12	24	36	48
Household Environment and Demographics	✓	✓	✓	✓	✓	✓
**CHILD**						
Oral Hygiene Behaviours		✓	✓	✓	✓	✓
Clinical examination		✓	✓	✓	✓	✓
Child general health & temperament		✓	✓	✓	✓	✓
Oral Health Related Quality of Life					✓	✓
Food Frequency Questionnaire		✓	✓	✓	✓	✓
Body Mass Index		✓	✓	✓	✓	✓
Fluoride Levels (toenail clippings)			✓	✓	✓	✓
**PARENT**						
Oral Hygiene Behaviours	✓	✓	✓	✓	✓	✓
Self-reported oral health status		✓			✓	
Parent mental health & family functioning	✓	✓	✓	✓	✓	✓
Food Frequency Questionnaire		✓			✓	
Fluoride Levels (Household Water Supply)				✓		✓
Attitudes and Choice (Beverage consumption; DCE)			✓	✓	✓	

### Participants and Recruitment

We will recruit 500 expectant mothers and retain the parent-child dyads over this five-year study (child age 0 to 4 years). With an anticipated attrition of 2% of the sample each year there will be 450 participants remaining at child age 4 years. A sample of 450 participants will provide the power to detect a 3.6% change in the prevalence of rare outcomes (eg caries) with 80% power at a significance level of 0.05, or a change in prevalence by 6.6% with 80% power at a significance level of 0.05 for outcomes prevalent at 50% in the sample. Participants will be recruited from across the Barwon-South Western region of Victoria, Australia. This region is one of eight regions in Victoria and has an estimated population of 350,109. The region includes Geelong (population over 200,000) as the regional centre and comprises rural and regional geographical contexts. It is socio-economically disadvantaged when compared to State-wide averages (including lower average income and employment status) and in 2006 15.8% of the population were born overseas[27].

Expectant mothers will be recruited during any gestational stage at antenatal clinics of major public hospitals and general practitioner clinics. Expectant mothers who agree to participate will provide written consent, complete a baseline questionnaire and be contacted again approximately three to four months after the birth of their child. A small number of key demographic questions will be asked of expectant mothers who choose not to participate in the study to allow for assessment of participation bias.

#### Inclusion and exclusion criteria

Inclusion criteria are that the adult participant is the primary carer of the child participant, lives in the Barwon-South Western region of Victoria and speaks English. If over the course of the study, the child's primary carer changes, the new primary care giver will be invited to participate in the study. There is a possibility that participants may experience pregnancy or birth complications that could lead to the loss or ill health of their infants. Because of the sensitive nature of this subject, information about participants' hospital discharges will be ascertained through hospital records between baseline data collection (antenatal) and contacting participants for follow-up data collection (when the infant is 6 months of age). Participants who were not discharged from the hospital with an infant will not be contacted.

### Measures and Procedures

#### Qualitative interview

A sub-group of parents (n = 20-40) will be purposively selected on the basis of responses to the initial questionnaire and invited to participate in the qualitative study of parental choices concerning children's beverage consumption. The sampling will attempt to include as diverse a sample as possible, with diversity in geographical location, type of household water consumed (tank, tap, bottled, etc.), socioeconomic status (education, income, etc.) and number of children in the household used as the primary selection variables. Interviews will continue until data saturation (no new information gathered from interviews) is reached[28].

The interviews will be semi-structured and follow a topic guide which will allow for changes in the direction of the interview based on a participant's response to topics covered. The topic guide will include items on perceptions about the palatability and safety of tap water, water restrictions and water targets for the area in which they live, participant's child feeding practices and the factors that influence participant's drink choices. Towards the end of the interview, the participant will also be asked a series of more structured questions to reflect on the most important features for them in determining drink choice, and whether the price of different drink options is an important consideration. This approach has been used previously and found to be feasible and acceptable to participants and interviewers and often proved to be a valuable tool with which to summarise the key points of the interview and close the interview in a positive way. All interviews will be audio-recorded and transcribed verbatim by a trained researcher. The transcripts will be compared to the audio-recordings to ensure accuracy. The data from the more structured section of the interview will be used to define attributes of the discrete choice experiment included in the study questionnaire. All the data from the interviews will be used to inform the design of subsequent parent questionnaires.

#### Questionnaires

Participants will be asked to complete questionnaires prior to the birth of their child and when the child is aged six, 12, 24, 36 and 48 months. Questionnaires will include items across several domains, such as socio-economic status and demographics, household water environment (source of water, use of filters, etc.), accessibility of different types of beverages, history of fluoride exposure, dietary and non-dietary sources of fluoride, oral health care, use of general and oral health care services, dental treatments, and general and oral health status. Many items to be included in these questionnaires have been used previously in other epidemiological research [29-31]. Questionnaires at 24 and 48 months will include a Discrete Choice Experiment (DCE) to assess the relative importance of different factors influencing drink choices and changes in the relative importance of these factors across socio-economic groups.

##### Household data, Demographics, Water Environment, Attitudes and Health Status

These data will be collected via a written parental questionnaire at recruitment (i.e. before child is born) and will include items to assess participant socio-economic status and demographics including number and age of household members, parental education, occupation and household income (standardised and validated items from routine data collections and previous studies [21,29,30]. The questionnaire will also contain items on water sources available to the household (e.g. mains or tank water, use of filters) restrictions on water use, and on physical accessibility and financial cost of alternative household beverage consumption options. Parental attitude data will be collected using Likert-scale responses to a series of positive and negative statements covering alternative water and non-water beverage consumption options, preventative oral health care and restorative dental treatment. Parents will also be asked to self-report their use of general and oral health care services, history of fluoride exposure, dental treatment and general health and oral health status using existing measures [30,33].

When the child is 6 months old, parents will be sent a second written questionnaire containing a repeat of items on household water environment (water sources, restrictions, etc.), parental attitudes and parental general and oral health status. The questionnaire will contain new items to assess parent-reported child general and oral health and child's history of health service use [32,21]. Parents will also be asked to report any change in household status (such as employment) and any use of health care services since the previous report. Parents will be asked to complete a third and fourth written questionnaire prior to the child's dental examination at 12, 24 and 48 months. These will repeat the format of the 6-month questionnaire, with items of household water environment, parental attitudes, parental and child general and oral health status and use of health care services.

##### Health data

parental and child general health status will be assessed via parental report in the written questionnaires at recruitment (for parental health) and at each time point (Table 1). Parents will also be asked at recruitment to provide consent for the study researchers to collect data from the child's Child Health Record ("Blue Book"). This record is kept by parents and contains data on birth outcomes (recorded by hospital midwife during the postnatal hospital stay), child developmental outcomes (recorded by the Maternal and Child Health (MCH) nurse at each of 8 Key Age and Stage (KA&S) visits and immunisation data (recorded by medical staff providing immunisation). Parents will be asked to bring their child's record with them to each of the dental examinations, where a researcher will transfer child health outcome data collected in the record to that date, including child weight and length/height. At the first dental examination this will include birth outcome data, immunisation status and child development data from a possible seven KA&S visits (two weeks, four weeks, eight weeks, and 4, 6, 12 and 18 months). At the second dental examination only additional data recorded since the initial examination will be collected (one or two KA&S visits at 2 and 3.5 years).

##### Anthropometry

Children will have their weight and length/height recorded at the 8 KA&S visits as outlined above. Anthropometric data are written into the child health record by the MCH nurse and these data have previously been found to be of high quality and useful for evaluation purposes[34].

##### Dietary measures

The Iowa Fluoride Study (IFS) beverage questionnaire will be used for the present study. The questionnaire has been validated against 3-day dietary diaries and found to provide a good estimate of fluoride and beverage intakes in children aged 6 months to 5 years [35]. The researchers in the Iowa Fluoride Study developed the beverage questionnaire to reduce participant burden (compared to completing 3-day food diaries) and to increase participant compliance with the data collection required during this long term, longitudinal study with frequent data collection points.

##### Discrete Choice Experiment (DCE)

The DCE method originates from marketing and psychology, but is firmly based in economic theory of choice. Factors influencing the choice of different types of drinks are first identified from the interviews and literature (e.g. prices, sugar content etc) and levels attached to each attribute [36]. Participants are then presented with a number of hypothetical alternative drinks, involving different combinations of attributes and levels, and for each choice asked which drink they would choose. From this it is possible to estimate: the effect of changes in the levels of different attributes on the probability of choosing one type of drink over another; how individuals trade between these attributes (i.e. how much of one attribute they are willing to give up to have more of another attribute); and willingness to pay for different attributes (including willingness to pay for different combinations of attributes and levels.

##### Fluoride Exposure

Potential sources of fluoride are dietary (beverages; mainly water, and foods) and fluoride containing oral health products (eg toothpaste).

a)*Dietary sources of fluoride: *Information on fluoride exposure from dietary sources will be collected via questionnaires (including water use) which will utilise brief items and scales that have been shown to be reliable and valid indicators of the variables of interest and reported in previous related research [37].

*b)Water sources of fluoride: *At each of two time points (Table 1), participants will be asked to provide a sample of water from their household tap and from a source other than the household tap if their child is primarily ingesting this water rather than tap water. Water samples will be analysed for fluoride content and to estimate the child's fluoride intake from water, the fluoride concentration of the child's main water source will be multiplied by the parent-reported child's usual water intake (approximated through parental questionnaire). Similar methods have previously been used to estimate young children's fluoride intake from water [38-40].

c)*Non-dietary sources of fluoride: *Measures of non-dietary ingestion will be estimated using five items that have been pre-tested in the VCHWS [21] and by asking parents to record the amount of fluoride toothpaste products using techniques also employed in the IFS [41,42]. It is unlikely that fluoride supplements or mouth rinses will be used but this information will be recorded and total ingestion estimated from parent reports of dosages.

#### Biomarker measurement

At several time points (Table 1), participants will be asked to collect their child's toenail clippings for subsequent analysis of fluoride concentration. Participants will be asked to refrain from cutting their child's toenails for at least two weeks prior to collecting these clippings, which is common methodology for collecting toenail clippings [43-45]. The fluoride content of toenail clippings of young children has been found to be a suitable biomarker of both sub-chronic [45] and chronic [46] exposure to fluoride. Toenail clippings rather than fingernail clippings, will be collected as they have lower exposure to environmental fluoride than fingernail clippings [43,44].

##### Oral Health: Dental examination

Children will undergo a dental examination at a community (public) dental clinic when the child is 12, 24 and 48 months of age. Dental examiners will use the International Caries Detection and Assessment System (ICDAS) II to assess all tooth surfaces (distal, occlusal, mesial, buccal and lingual) of each tooth present. ICDAS is a visually based diagnostic system that was developed as a result of a systematic review of clinical caries detection systems [47-49]. The system is intended for use by clinicians, epidemiologists and researchers for standardised data collection in various settings and improved comparison between study results [50]. The most recent version, the ICDAS II examination procedure, is similar to that of the WHO basic methods for conducing oral health surveys [51], which has been traditionally used in epidemiological research [52]; however, the ICDAS II system allows for the detection of non-cavitated carious lesions that would not be detected by the WHO assessment method. The ICDAS II system has demonstrated reproducibility and diagnostic accuracy for occlusal caries detection [49,50], which is particularly important considering that the most commonly caries-affected sites for children are the occlusal tooth surfaces [49,50]. ICDAS II has been reported to be feasible for use in epidemiological surveys in preschool children [53], and a modified version of the method is currently being used in other epidemiological studies conducted with 4- to 18-month-old children[29].

### Analysis

#### Qualitative data

Data coding and thematic analysis will be conducted alongside the collection of qualitative data from interviews to allow for ongoing analysis and refinement of attitude grouping until data saturation is reached. Interview transcripts will be coded by the study investigators and techniques in inductive theory, specifically the iterative process of open coding and grouping of similar codes will be used to extract the issues emerging. Qualitative data will be organised using NVivo software.

#### DCE analysis

Choice data from the DCE will be analysed using standard econometric techniques [54]. The DCE will be analysed using a mixed logit regression model, where the dependent variable is whether the respondent chooses Drink A or Drink B, and independent variables are the attribute levels in each alternative. Mixed logit is now the standard analytic approach for DCEs and helps to account for a number of sources of unobserved heterogeneity to produce unbiased regression coefficients [54]. The sample size of 500 parent-child dyads will be sufficient for the analysis of choice data by main sub-groups, such as socio-economic group and geographic location

#### Longitudinal study analysis

The longitudinal data will be analysed using regression-based statistical and econometric methods. A sequence of regression equations will be estimated that account for the relationships between three variables: primary outcome measures (dmft and standardised body mass index), fluoride exposure, and tap water consumption. To begin with, each of these will be modelled in separate regression models. Mediating and moderating variables will be explored and will likely include family demographics and composition, water environment, diet, and consumption of alternative drinks. Additional models will test the relationship between tap water consumption and fluoride exposure on the primary outcomes. A mixture of categorical and continuous variables will be used. Variables will be first differenced to examine the change in the dependent variable between time period 1 and time period 2, as a function of the change in the levels of independent variables between time periods 1 and 2. This has the advantage that all factors that do not change over time, including those which cannot be observed, are equal to zero for each respondent and therefore are controlled for in the analysis [55]. This will include factors about geographic location, genetic influences on health, and socio-economic factors. This provides more unbiased estimates of the effect of fluoride exposure, drink choices, and water environment on the primary outcomes compared to using cross-sectional (contemporaneous) data. In addition, regression models will be estimated using values of each dependent variable at time period 2, regressed on past levels of the independent variables (time period 1). This removes the effect of reverse causality and further reduces bias. Survival analysis techniques will also be utilised.

##### Ethics approval and consent

The study has been approved by the University of Melbourne Human Research Ethics Committee and the Barwon Health Human Research Ethics Committee. All participants will provide written informed consent prior to enrolling in the study for themselves (mother) and their child.

## Discussion

This study aims to make a significant contribution to the evidence base for a number of national and international public policy priorities including community water fluoridation, drought, child obesity and dental caries. These issues and their associated policies contribute to increasing health and social inequalities via their impact on multiple outcomes (social, economic, education and health). This will also be the first Australian (and possibly international) study to disentangle the relationship between beverage consumption and the effectiveness of community water fluoridation. It is an essential contribution to the social and public health policy context given that the public health intervention of community water fluoridation is unlikely to be effective in rural and regional communities if consumption of fluoridated tap water is low.

Developing the knowledge base of what influences people's consumption of tap water and other water is essential in the planning of population-wide prevention strategies for early childhood caries and obesity. The research will therefore include an explicit assessment of the role of factors influencing drinks choice, including beverage quality and the relative prices of drinks. The impact of drinks choice on the effectiveness of water fluoridation in reducing socio-economic inequalities in health will also be examined. Further, the analysis will also recognise that water fluoridation influences drinks choice ('reverse causality') which will be examined through the prospective, longitudinal study.

A mix of innovative research methods will also be used. From health economics, this includes DCE and the econometric analysis of discrete choices and longitudinal data combined with rigorous qualitative and epidemiological methods. Specifically, the DCE presents individuals with a range of hypothetical choices that vary according to pre-defined attributes. This is a novel application of this methodology into public health, but is ideal for studying the relative importance of factors influencing drinks choices and how the importance of these factors varies across different socio-economic groups. Combining the DCE data with actual consumption data from the longitudinal survey is a particular innovation that can be used to predict changes in the consumption of drinking water as a result of policy change. Furthermore, the use of prospective longitudinal data helps remove certain sources of bias to produce more accurate estimates of the effects of interest compared to using cross-section or historic data.

The novelty and innovation of this research is related to the combination of mixed methods and study types with an integrated chronic disease prevention framework[25]. Thus, this study involves a social science exploration of what parents think, why they think the way they do and how they might change their consumption if the choice factors changed, combined with a longitudinal dental health and nutrition study of what families consume over time and how this relates to their level of (and changes in) dental and general health. Conventionally these studies are disconnected and to our knowledge have not been conducted previously within the same population.

In conclusion, this study will have a direct policy impact and potential for generalisability to other populations and similar contexts. The study is linked closely to a range of water policy issues such as desalination, recycling, and other infrastructure such as long pipes that transcend government boundaries. A key issue is the effect of changes in water supply and its production and consumption on health status which, until now, has not been investigated. The results of the study will be used by government and government agencies to influence prevention policies, social marketing, and environmental programs in health and water. The expected economic, environmental and social benefits to the wider community are improved health, reduced use of hospital emergency services, reduced demand on stretched public dental service, reduced incidence of obesity and diabetes, improved social, learning and educational outcomes, and increased productivity.

## Competing interests

The authors declare that they have no competing interests.

## Authors' contributions

EW, AdS, MG, AS, LG, MS and HC conceptualized the study and secured funding for the study. All authors were involved in determining the study methodology and KL, MV-M and AdS drafted the initial manuscript. All authors reviewed versions of the manuscript and approved the final version.

## Pre-publication history

The pre-publication history for this paper can be accessed here:

http://www.biomedcentral.com/1471-2458/11/505/prepub
